# The inter-relation between policy and practice for transitions from hospital to home: an ethnographic case study in England’s National Health Service

**DOI:** 10.1186/1472-6963-14-S2-P111

**Published:** 2014-07-07

**Authors:** James Shaw, Pia Kontos, Wendy Martin, Christina Victor

**Affiliations:** 1 Toronto Rehabilitation Institute, Toronto, Canada; 2 Brunel University, London, UK

## Background

Poorly managed transitions from hospital to home for elderly patients with complex needs (commonly referred to as patient discharge) often exacerbate detriments to health and function associated with acute inpatient hospital stays. The English National Health Service introduced the Community Care (Delayed Discharges) Act in 2003 to improve the transition process, but the ways in which this policy is interpreted and applied in actual contexts of health and social care remain under-explored using qualitative methodologies. The purpose of this study was to understand the complex interrelationship between policies related to discharge from hospital and the practice of helping older people transition from hospital to home in London, United Kingdom.

## Materials and methods

We used an ethnographic case study methodology to achieve a comprehensive understanding of the relationship between policy and practice for patient transitions, including the following methods: (a) observation of inter-professional team meetings in the hospital and community settings, (b) qualitative interviews with key informants including health care leaders, commissioners of care, practitioners, and patients, (c) analysis of policies at the national and local levels related to patient transitions, and (d) patient chart reviews. Data were analyzed from a critical social science perspective, and key themes were related between data sources. An overall understanding of the process of policy implementation for patient transitions were then interpreted.

## Results

Findings suggest that the implementation of policies for patient transitions relied on informal practices and relationships at the individual, organizational, and inter-organizational levels as opposed to formal mechanisms of control embedded in national policy (e.g., financial penalties for poor transitions). At the individual level, health care practitioners negotiated new mandates related to patient transitions in the context of existing informal relationships related to patient care decision-making. At the organizational level, health care managers relied on motivating and encouraging improved practice among staff as opposed to enforcing strict adherence to policy guidelines. At the inter-organizational level, healthcare leaders built informal relationships with those at partner organizations in order to build trust and encourage collaboration during the process of patient transitions.

## Conclusions

This work suggests that greater attention should be paid to (a) the personal characteristics of health care leaders and practitioners and (b) the conditions of practice in health care environments that help to foster relationship-building in the context of patient transitions from hospital to home. These informal relationships were found to be central to policy implementation for patient transitions.

